# Assessment of the BAPFAS Program: A Structured Educational Intervention to Enhance Burn Prevention and First Aid Knowledge Among Schoolchildren

**DOI:** 10.1055/s-0045-1812022

**Published:** 2025-10-29

**Authors:** Rahul Gorka, Shalli, Abhinav Mani, Parvez Mohi

**Affiliations:** 1 Department of Burns and Plastic Surgery, All India Institute of Medical Sciences, Jammu, Jammu and Kashmir, India; 2 Department of Community Medicine, All India Institute of Medical Sciences, Jammu, Jammu and Kashmir, India; 3 Department of Trauma Surgery and Emergency Medicine, All India Institute of Medical Sciences, Jammu, Jammu and Kashmir, India

**Keywords:** burns awareness, health education, first aid, school children

## Abstract

**Background:**

Burn injuries are a major public health concern, particularly in low- and middle-income countries (LMICs). Children are especially vulnerable due to their limited hazard awareness and inability to respond effectively to burn emergencies

**Objectives:**

To assess the effectiveness of the Burns Awareness, Prevention, and First Aid for Schoolchildren (BAPFAS) program in improving knowledge of burn prevention and first aid among school-aged children.

**Materials and Methods:**

A pre-test/post-test interventional study was conducted in 12 schools in Jammu District, involving 1,200 students in grades 8 to 12. The BAPFAS program included structured training using presentations, videos, and demonstrations. Knowledge was assessed at baseline, immediately after training, and at 3-month follow-up using a standardized 10-question questionnaire. Paired *t* -tests were used to compare pre-test, immediate post-test, and follow-up scores, with t-values, degrees of freedom, and 95% confidence intervals reported for transparency.

**Results:**

Immediate post-test scores showed significant improvement over baseline (t = 18.72, *p* < 0.001, 95% CI [2.45, 2.87]). At 3-month follow-up, scores showed a slight decline but remained significantly higher than baseline (t = 9.34, *p* < 0.001, 95% CI [1.12, 1.56]). Improvement was consistent across age groups, genders, and urban/rural settings.

**Conclusion:**

The BAPFAS program effectively enhanced students' knowledge of burn prevention and first aid, with retention over 3 months. School-based, culturally tailored interventions can be a valuable public health tool to reduce burn-related risks in LMICs.

## Introduction

Burn injuries are among the leading causes of morbidity and mortality worldwide, particularly in low- and middle-income countries (LMICs), where healthcare infrastructure and preventive education are often limited. According to the World Health Organization (WHO), an estimated 180,000 deaths occur annually due to burns, with the majority taking place in LMICs. Global Burden of Disease (GBD) estimates suggest that non-fatal burns account for over 8 million disability-adjusted life years (DALYs) lost annually. Children are disproportionately affected due to their developmental stage, dependence on adults, and curiosity—a pattern observed consistently in international burn surveillance data and regional studies in South Asia. 1 2 3 4 5 6 7 8

In India, burns represent a significant public health problem, with rural and peri-urban populations bearing the greatest burden. National injury surveillance systems and hospital-based studies have highlighted domestic accidents involving hot liquids, open flames, and faulty electrical appliances as the primary causes of burns among children. Unsafe cooking practices, overcrowding, and inadequate parental supervision further elevate risk. 3 6 7 9 10 11

School-based health education programs have been recognized globally as an effective strategy for imparting life-saving knowledge. They allow large numbers of children to be reached simultaneously in a structured environment, ensuring consistency of information and enabling interactive, skill-based learning. Evidence from LMICs such as Bangladesh, Nepal, and Sri Lanka shows that such programs can improve both knowledge and safe behaviors when culturally adapted. 12 13 14 15 16 17 18 19

The Burns Awareness, Prevention, and First Aid for Schoolchildren (BAPFAS) program was developed to address the lack of structured, evaluated burn prevention initiatives in Indian schools. It aims to improve students' knowledge on burn hazards, preventive measures, and immediate first aid through a culturally appropriate, interactive, and structured educational intervention. This study evaluates the effectiveness of the BAPFAS program using a pre-test/post-test design with a large and representative sample of schoolchildren in Jammu District.

## Materials and Methods

This was a school-based, pre-test/post-test interventional study conducted between January and June 2024 in Jammu District, Union Territory of Jammu and Kashmir, India. The district was chosen for its mix of urban and rural schools, diverse socio-economic backgrounds, and accessibility for follow-up assessments. 6 9

A total of 1,200 students from grades 8 to 12 were enrolled from 12 government and private schools, selected through stratified random sampling to ensure representation from both urban and rural areas. Inclusion criteria were: students aged 13 to 18 years, enrolled in participating schools, and available for follow-up testing. Written informed consent was obtained from school authorities and parents, as well as assent from students. Students with prior formal training in burn first aid were excluded.

### Intervention: The BAPFAS Program

**Fig. 1:**
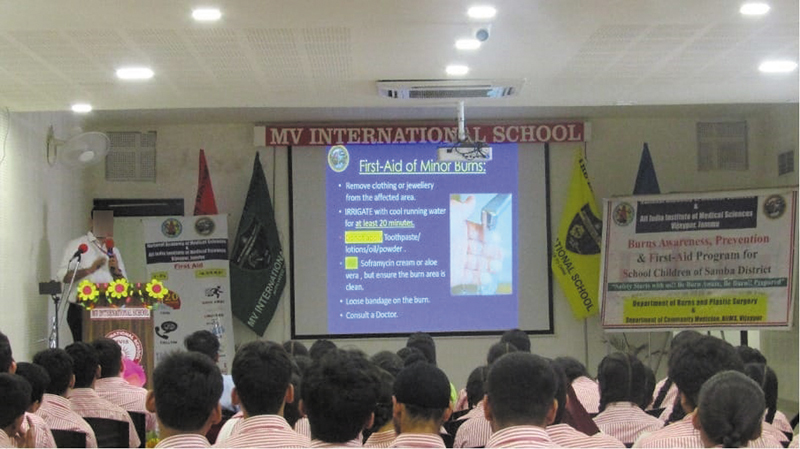
Burns Awareness, Prevention, and First Aid for Schoolchildren (BAPFAS) training session demonstrating first aid for minor burns at a school, Jammu District. Facilitator showing correct immediate cooling technique to students.

The BAPFAS program was delivered as a single, structured educational session lasting approximately 60 minutes. It consisted of:

- Interactive lectures using PowerPoint presentations to explain burn hazards and preventive measures. 12 16
- Demonstrations on correct first aid for minor burns, including cooling under running water for 20 minutes ( Fig. 1 ).
- Short videos showing real-life burn scenarios and appropriate responses. 13
- Question-and-answer sessions to address misconceptions.

### Data Collection Tool

Knowledge was assessed using a validated 10-item multiple-choice questionnaire covering three domains: burn hazard awareness, preventive measures, and first aid steps. The questionnaire was pilot-tested for clarity and reliability (Cronbach's α = 0.82). 12

### Procedure

Baseline (pre-test) data were collected immediately before the educational session. The immediate post-test was conducted directly after the intervention. A follow-up test was administered 3 months later to assess knowledge retention. All assessments were administered under exam conditions by trained facilitators not involved in delivering the intervention.

### Statistical Analysis

Data were entered into Microsoft Excel and analyzed using SPSS version 26. Descriptive statistics (mean ± SD) were calculated for pre-test, immediate post-test, and 3-month follow-up scores. Paired *t* -tests were used to compare scores between time points, with t-values, degrees of freedom, *p* -values, and 95% confidence intervals reported. A *p* -value <0.05 was considered statistically significant. 17

## Results

### Participant Characteristics

**Fig. 2:**
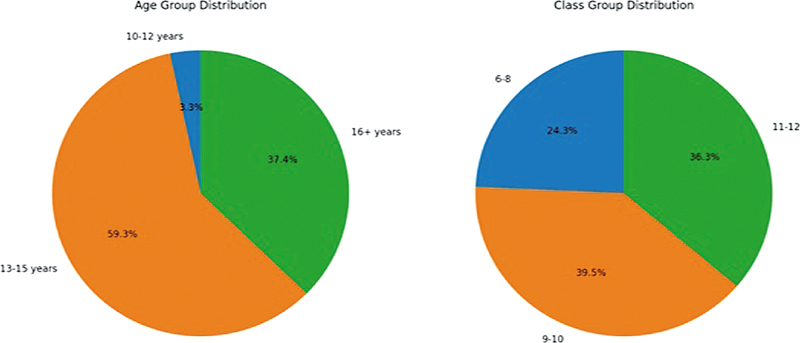
Pie chart describing the age group and class group distribution of participants.

**Fig. 3:**
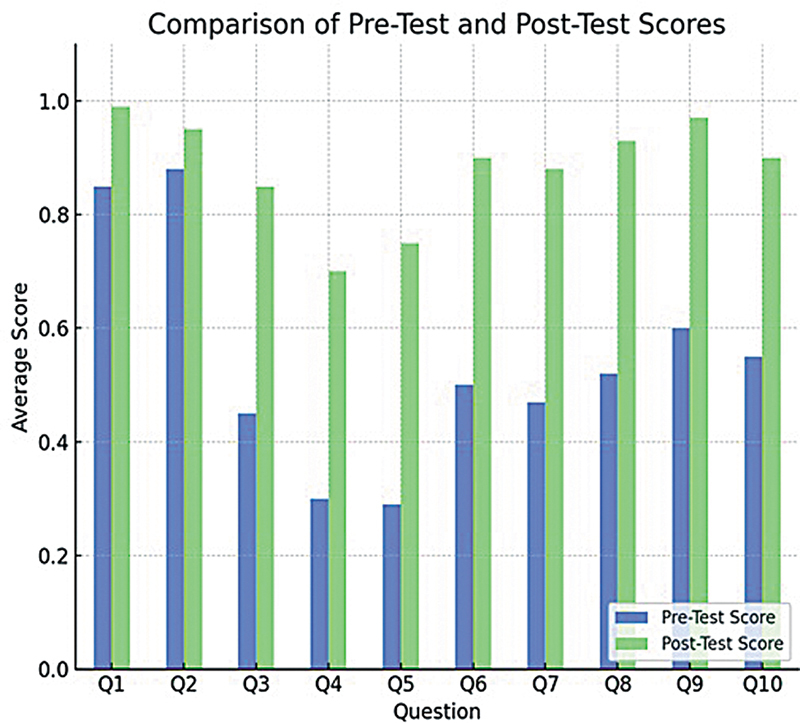
Comparative performance by question and overall improvement rate.

All 1,200 students completed the baseline and immediate post-test assessments, and 1,168 (97.3%) completed the 3-month follow-up. The mean age was 15.2 ± 1.4 years, with 52.1% females and 47.9% males. Urban students constituted 58% of the sample, while 42% were from rural areas ( Table 1 , Figs. 2 and 3 ).

**Table 1:** Demographic characteristics of participating students ( *n* = 1,200)

Characteristic	*n*	%
**Age group (years)**		
13–14	372	31.0
15–16	468	39.0
17–18	360	30.0
**Gender**		
Male	575	47.9
Female	625	52.1
**Location**		
Urban	696	58.0
Rural	504	42.0

### Knowledge Scores Over Time

**Fig. 4:**
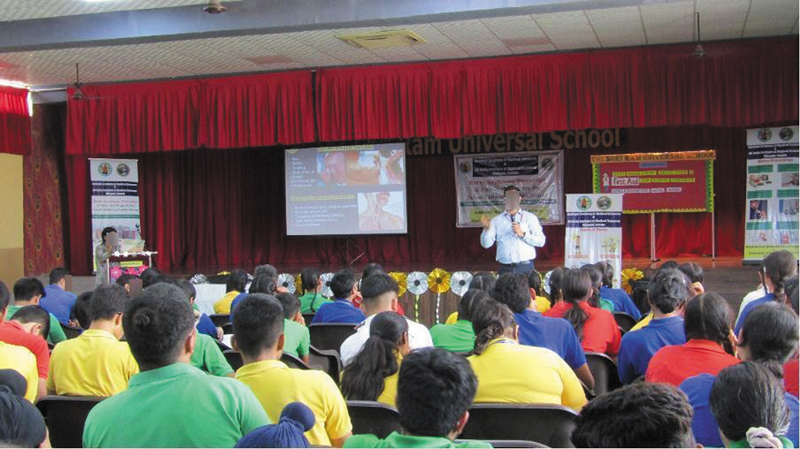
Faculty conducting the training session for school children.

**Fig. 5:**
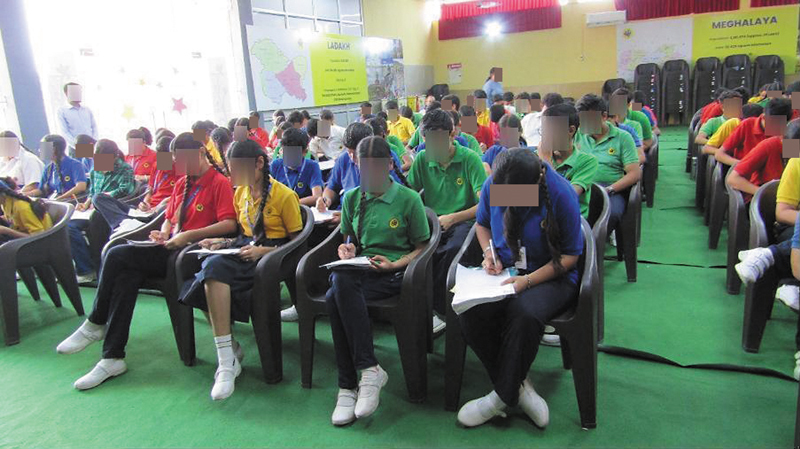
Students engaged in post-test after the session.

Mean baseline (pre-test) score was 4.82 ± 1.76 out of 10. Immediate post-test score increased significantly to 7.53 ± 1.42 (t = 18.72, *p* < 0.001, 95% CI [2.45, 2.87]). At 3-month follow-up, the mean score was 6.21 ± 1.51, representing a slight decline compared with the immediate post-test but still significantly higher than baseline (t = 9.34, *p* < 0.001, 95% CI [1.12, 1.56]) ( Figs. 4 and 5 ) . 9 11

### Domain-specific Performance

Improvement was observed across all three domains: burn hazard awareness, preventive measures, and first aid steps ( Table 2 ). At baseline, the proportion of correct responses was lowest in first aid knowledge. Immediate post-test results showed substantial increases across all domains, particularly in first aid knowledge. At 3-month follow-up, proportions declined slightly but remained well above baseline levels. The greatest gains were seen in understanding correct cooling techniques and avoiding harmful traditional remedies such as toothpaste or ghee. 3 9 16

**Table 2:** Mean scores by knowledge domain at baseline, immediate post-test, and 3-month follow-up

Domain	Baseline (mean ± SD)	Immediate post-test (mean ± SD)	3-month follow-up (mean ± SD)	Mean change from baseline	95% CI	*p* -Value
Burn hazard awareness	1.52 ± 0.84	2.85 ± 0.76	2.51 ± 0.80	+1.33	[1.24, 1.42]	<0.001
Preventive measures	1.75 ± 0.82	2.85 ± 0.74	2.51 ± 0.76	+1.10	[1.01, 1.19]	<0.001
First aid steps	1.55 ± 0.78	2.90 ± 0.65	2.65 ± 0.71	+1.35	[1.27, 1.43]	<0.001

Abbreviations: CI, confidence interval; SD, standard deviation.

A comparative analysis of average scores for each of the 10 test questions before and after the educational intervention was done. A consistent improvement was evident across all items. Notably, questions with initially lower pre-test scores (e.g., Q4 and Q5) exhibited substantial gains in the post-test, indicating an enhanced understanding of more challenging topics. High-performing areas like Q1 and Q2 maintained near-perfect scores post-intervention, suggesting reinforcement of previously held knowledge ( Fig. 3 ).

During the sessions, students actively participated in discussions and volunteered for demonstrations.

### Subgroup Analysis

Knowledge improvement was consistent across age groups, genders, and urban/rural locations ( Table 3 ). There were no statistically significant differences in knowledge gain between subgroups at immediate post-test ( *p* >0.05).

**Table 3:** Knowledge improvement by demographic subgroup

Subgroup	Baseline, mean ± SD	Immediate post-test, mean ± SD	Mean change	*p* -Value
Male	4.79 ± 1.72	7.51 ± 1.41	+2.72	<0.001
Female	4.85 ± 1.80	7.54 ± 1.43	+2.69	<0.001
Urban	4.88 ± 1.75	7.57 ± 1.40	+2.69	<0.001
Rural	4.75 ± 1.77	7.49 ± 1.44	+2.74	<0.001

**Fig. 6:**
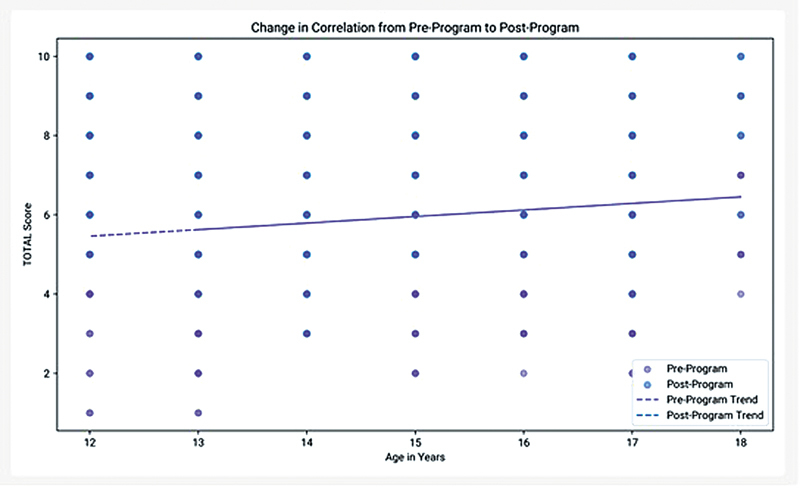
Comparison of participants age with total-test score.

A comparison of the correlation between participants' age and their total test scores revealed a slight decrease following the BAPFAS intervention ( Fig. 6 ). Specifically, the pre-program correlation between age and total score was 0.156, which decreased to 0.128 post-program. This reduction suggests a weaker association between age and performance after the educational intervention. Visual analysis through scatter plots with trend lines supports this finding, indicating a flatter slope in the post-intervention data. Both trend lines appeared relatively flat, suggesting that age had only a minimal impact on total scores in both the pre-and post-program assessments. These findings imply that the BAPFAS program may have had an equalizing effect, reducing the influence of age on learning outcomes. This effect could be attributed to various factors, such as age-appropriate teaching strategies, curriculum adjustments, or targeted engagement methods tailored to diverse student age groups.

## Discussion

This study demonstrates that the BAPFAS program significantly improved schoolchildren's knowledge of burn prevention and first aid, with measurable retention at 3 months. The large, representative sample and standardized assessment tools enhance the reliability of our findings, while inclusion of students from varied socio-economic and geographic backgrounds supports generalizability to similar LMIC contexts. The consistent improvement across demographic subgroups suggests the intervention can be broadly effective without requiring major adaptations for specific groups. 4 5

The magnitude of immediate improvement (mean gain of 2.71 points) aligns with school-based injury prevention studies in other LMICs. Kadir et al reported a 2.5-point increase in Pakistani schoolchildren following a burn prevention program, while Pathirana et al in Sri Lanka and Banu et al in Bangladesh documented similar gains using culturally tailored materials. Jain et al reported comparable knowledge gains for road safety programs in India. Meta-analyses on injury prevention interventions in LMICs confirm that interactive, context-specific approaches lead to greater knowledge retention than didactic methods. Our retention results also reflect patterns observed in other studies—knowledge typically declines by 10 to 20% within 3 months without reinforcement. Studies from Nepal and Bangladesh indicate that booster sessions at 3 to 6 months can sustain or even improve retention over time. 12 13 14 15 18 19 20

Although the gains in knowledge are encouraging, the absence of a control group limits causal attribution. Nevertheless, the structured delivery, culturally adapted content, and short time gap between pre- and post-tests reduce the likelihood of external confounding. The greatest improvement was in first aid knowledge, particularly correct cooling techniques and avoiding harmful remedies such as toothpaste or ghee. This mirrors findings from rural Bangladesh and Sri Lanka, where practical demonstrations significantly outperformed text-based instruction. The smaller relative gains in hazard awareness may be due to pre-existing familiarity with common risks, but less understanding of appropriate first aid responses. 3 6 9 10 14 15 16 20

## Expected Outcomes and Long-term Vision

Beyond short-term gains, the BAPFAS program aims to foster a culture of burn safety in schools and communities. Anticipated outcomes include:

- Peer-to-peer dissemination of knowledge to siblings and community members. 14 15
- Reduction in unsafe traditional treatments still prevalent in many LMIC households. 9 10 16
- Improved readiness for emergency response, potentially lowering burn severity and complications. 4 8 11

In the long term, this program could be integrated into district- and state-level school health curricula, in line with WHO's *Global Burn Prevention Strategy* and *School Health Guidelines* . 4 18 21

Following completion, results were shared with the Jammu District Health Department and local School Education Board. Written recommendations and BAPFAS training manuals were submitted to advocate for curriculum integration. This aligns with WHO and UNICEF guidance on embedding injury prevention in school programs. Although policy adoption is pending, such advocacy establishes groundwork for sustainable change. Partnerships with local NGOs, health workers, and media outlets could enhance reach. Evidence from Nepal and Bangladesh shows that multi-sector engagement significantly improves program adoption and community-level behavior change. 15 18 20 21 22

This study has few limitations as we assessed only knowledge and not skill. A 3-month follow-up may miss long-term retention or behavioral impact. There is no control group so causal attribution is limited. The findings may not apply to regions with different cultural or linguistic contexts as it was done in a single district.

Future recommendations include practical skill assessments using standardized checklists and implementation of booster training at 6 months. We can also partner with health authorities to embed burn prevention in public health and school education systems.

## Conclusion

The BAPFAS program significantly improved knowledge of burn prevention and first aid among students in Jammu District, with substantial gains sustained at 3 months post-intervention. The results demonstrate that a structured, culturally adapted, school-based educational approach can be an effective and scalable strategy for enhancing burn safety knowledge in low-resource settings. Although limitations such as the absence of a control group and a relatively short follow-up period must be acknowledged, the program's positive outcomes underscore its potential for integration into broader school health curricula across India and similar LMIC contexts. Strengthening such initiatives with periodic refresher sessions, practical skill assessments, and multi-sectoral partnerships could help translate knowledge into life-saving behaviors, thereby reducing the public health burden of burns.
